# Prolonged return to work and hampered work ability: insights from a scoping review on the impact of long COVID on healthcare workers job performance

**DOI:** 10.1186/s12913-026-15032-w

**Published:** 2026-06-30

**Authors:** Pia Delano, Vicky Serra-Suton, Fernando G. Benavides, Mireia Utzet

**Affiliations:** 1 Center for Research in Occupational Health (CiSAL), Department of Medicine and Life Sciences, Universitat Pompeu Fabra, Barcelona, Spain; 2 Center for Biomedical Research Network (CIBER) of Epidemiology and Public Health, Madrid, Spain; 3 Agència de Qualitat i Avaluació Sanitària de Catalunya (AQuAS), Barcelona, Spain; 4 Hospital Del Mar Medical Research Institute (IMIM), Barcelona, Spain

**Keywords:** COVID-19, Post-acute COVID, Occupational health, Healthcare, Long COVID

## Abstract

**Background:**

Healthcare workers (HCWs) may have a higher risk of developing long COVID due to greater exposure to COVID-19, with symptoms extending beyond the acute phase impacting their daily living activities and job performance.

**Aims:**

To systematically map the existing literature on the impact of long COVID on HCWs’ job performance, focusing on their time to return to work and work ability.

**Methods:**

A scoping review following PRISMA-ScR guidance included peer-reviewed studies in English or Spanish (January 2020–December 2024). Four databases were searched. Experimental, epidemiological and qualitative studies were eligible. Two reviewers independently screened records. Methodological quality was appraised using the Mixed-Methods Appraisal tool (MMAT).

**Results:**

Nineteen studies were included, mainly European and predominantly cross-sectional. Long COVID was most often defined as symptoms lasting ≥ 3 months. Among HCWs with prior infection or whole-staff samples, prevalence ranged from 10% to 74%, with multiple reports above 50%. Thirteen studies evaluated RTW, with 19–63% resumed work within six months, commonly with restrictions, while around one in five remained unable to work in some cohorts. Full-time employment decreased markedly (e.g., 57% pre-infection vs 31% at follow-up). Between 16% and 40% required workplace adjustments such as reduced hours, reassignment, or avoidance of night shifts. Sixteen studies reported diminished work ability compared with pre-infection or unaffected peers. Greater symptom burden, particularly cognitive impairment and fatigue, consistently predicted poorer outcomes. One study estimated a mean of 223 days to reach current work-ability levels. Older age, depression, comorbidity, and acute disease severity were recurrent associated factors while evidence for gender and job category was inconsistent.

**Conclusions:**

Long COVID delays RTW and reduces work ability in HCWs. Health services should plan long-term occupational follow-up, flexible reintegration pathways, and targeted accommodations while higher-quality longitudinal research refines risk and prognosis.

## Background

The COVID-19 pandemic has been one of the biggest challenges for public health worldwide, and one of the medium-term consequences has been the persistence of the symptoms beyond the acute infection phase [1]. Long COVID was defined on December 2021 by the World Health Organization (WHO) as “a condition that occurs in individuals with a history of probable or confirmed SARS-CoV-2 infection, usually 3 months from the onset of COVID-19, with symptoms that last for at least 2 months and cannot be explained by an alternative diagnosis. Common symptoms included fatigue, shortness of breath, cognitive dysfunction, but also others, and generally have an impact on everyday functioning” [1]. Additional studies, like the Spanish CIBERPOSTCOVID, similarly describe long COVID as a set of symptoms affecting quality of life and daily living activities [2], and having negative effect on the job performance of those affected [3, 4], making it a critical area for ongoing research. Reports reveal that the prevalence of long COVID varies significantly, with estimates ranging from 6.2% to over 70%, indicating challenges in identifying and measuring the condition consistently [1, 2, 5, 6].

Among those who could be particularly affected are healthcare workers (HCWs), who faced increased risk exposure in their workplace and report high prevalence rates of long COVID varying from 44.2% to 74.2% [7–13]. Beyond infection risk, HCWs have endured intense workloads, prolonged use of personal protective equipment, redeployment across services, moral distress, and chronic understaffing during the pandemic [14–16]. These conditions may exacerbate fatigue, complicate recovery, and create pressure to return to work despite persistent symptoms [17–19]. In addition, healthcare roles are often physically and cognitively demanding and closely linked to patient safety, meaning that even subtle functional limitations may substantially affect performance [20]. With long COVID affecting job performance in critical ways, such as delayed return to work (RTW) and reduced work ability (WA) [3, 4], understanding its impact on HCWs and the characteristics of their work (occupational group, shift work, etc) is crucial to ensuring workforce sustainability and effective healthcare delivery.

While several studies have examined long COVID’s impact on general job performance, few have focused specifically on HCWs, who are particularly susceptible to adverse outcomes due to high prevalence of presenteeism and workplace stress [20, 21]. Research suggests that long COVID impact on RTW and WA can relate to prolonged sick leave, reduced work schedules and even employment cessation in severe cases due to symptoms [3, 4, 22–26]. It has been estimated that one in ten people with long COVID stop working in the United Kingdom [3], while in the United States it was associated with unemployment and not working full time [27]. However, the evidence specific to HCWs remains fragmented. Studies often prioritize symptom prevalence or broad workforce implications rather than systematically examining how long COVID influences return to work, work ability, and the types of workplace accommodations that may facilitate recovery within healthcare settings. Differences in definitions, methodologies, and outcome measures further limit the ability of occupational health professionals and policy makers to derive clear and actionable guidance. To our knowledge, no previous review has comprehensively mapped these aspects in healthcare workers.

The aim of this scoping review is to map the existing literature on the impact of long COVID on HCWs’ job performance, focusing on RTW and WA. Specifically, this review seeks to 1) examine the evolution of the definition and reporting practices of long COVID in HCWs throughout the pandemic; 2) explore the prevalence, potential sociodemographic (e.g. age, gender) and occupational (e.g. occupation, shift) differences among HCWs affected with long COVID, and how these factors influence their job performance; 3) assess the impact of long COVID on HCWs’ job performance, particularly regarding RTW and WA; 4) identify strategies and measures implemented to reduce the impact of long COVID on healthcare worker’s job performance, including workplace accommodations and support mechanisms; and 5) highlight gaps in current research and practice regarding the impact of long COVID on HCWs’ job performance to guide future research and inform occupational health policies.

## Methods

The proposed scoping review was conducted in accordance with the Joanna Briggs Institute (JBI) methodology for scoping reviews, with eligibility criteria structured according to the Population-Concept-Context framework [28]. Key items have been reported following the Preferred Reporting Items for Systematic Reviews and Meta-Analyses extension for Scoping Reviews (PRISMA-ScR) [29]. The full protocol for the scoping review was registered in Open Science Framework in July 2023 (DOI: 10.17605/OSF.IO/AYHRB).

Population were HCWs who worked during the pandemic in the healthcare system, either in direct contact with patients or indirectly (technicians, administrative staff) within a healthcare organisation, from all countries and healthcare settings.

The concept of long COVID was guided by the WHO [1], CIBERPOSTCOVID [2] and, as the first published, the National Institute for Health and Care Excellence (NICE) definition: “Persistent signs and symptoms after an acute SARS-CoV-2 infection that cannot be justified by an alternative diagnosis are classified into either long-COVID (four weeks or longer) or post-COVID (more than 12 weeks)” [30].

The concept of job performance was guided by work ability (daily and cognitive functioning at work) and return to work (return to the preinjury or modified job, either fully or partially) [31, 32]. Work ability is commonly measured using the Work Ability Index (WAI) which considers the worker’s health status, demands of work, and resources [33].

The context was any healthcare setting internationally during the COVID-19 pandemic, including hospitals, primary care and long-term care.

The included studies were experimental, epidemiological and qualitative studies in peer-reviewed journals. Other reviews references were searched to identify additional studies not included in the original search. As this is a new condition, and there has been emerging misinformation regarding COVID-19, grey literature was excluded to ensure the inclusion of peer-reviewed information.

Exclusion criteria included research in populations not based on healthcare professionals, studies without any job-related variable, post-mortem study of COVID-19 patients, and studied related to COVID-19 vaccine antibodies.

The search strategy was based on identified keywords and index terms adapted for four bibliometric databases: MEDLINE, Web of Science, Cochrane library, and Scopus. (See ANNEX 1)

The initial search analysed the text words “long COVID”, “healthcare workers’’ and “job performance” in the title and abstract of the articles. A second search, from October 2022 to June 2024, followed the keywords of interest and index terms in all included bibliometric databases. The reference list of all included sources was screened for additional studies. Studies published since January 2020 in English and Spanish were included.

**Fig. 1:**
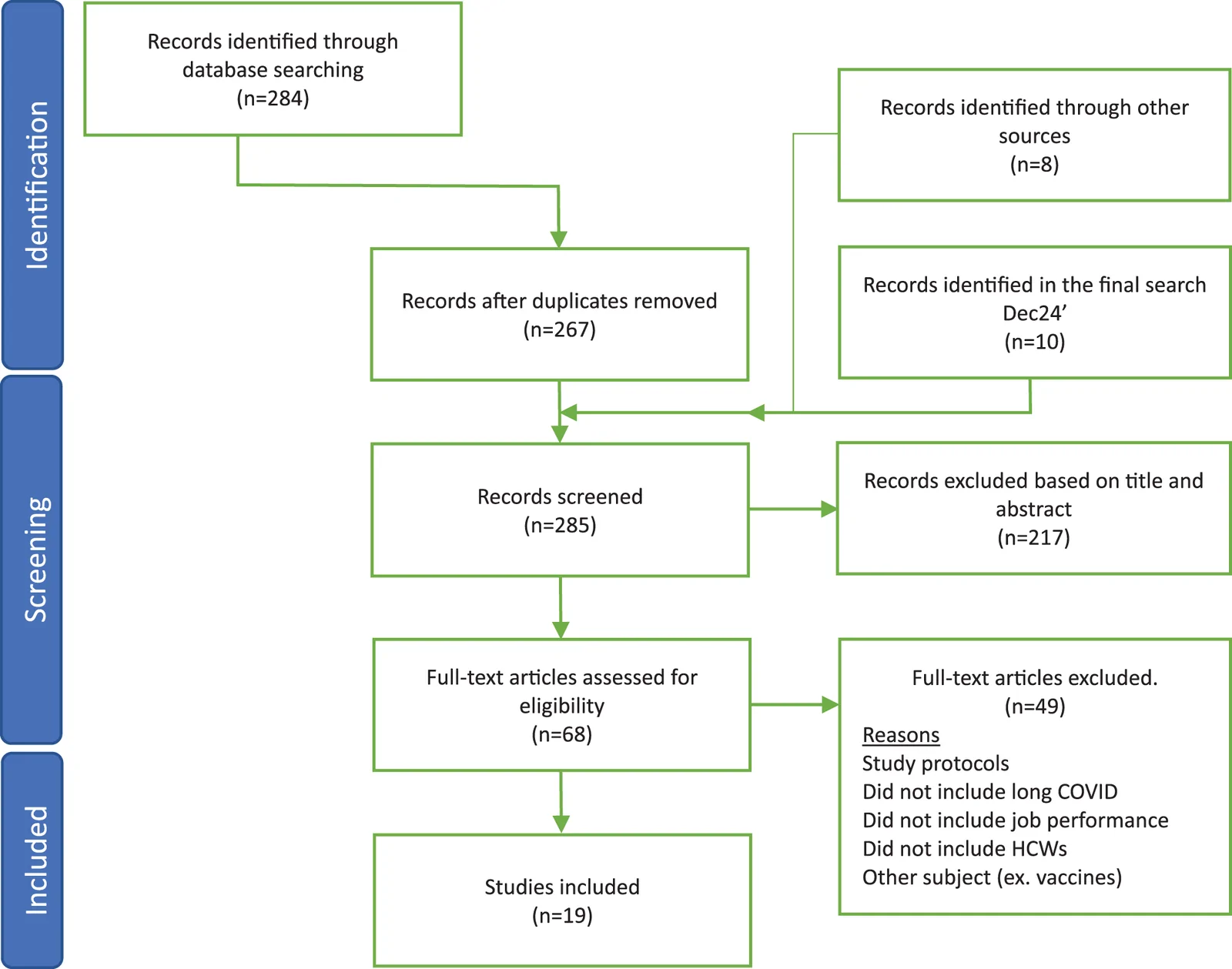
PRISMA-ScR flow diagram for scoping review of job performance in healthcare workers with long COVID

Results were cross referenced with Rayyan to delete duplicates [34]. Articles obtained through other sources were then added. The selection of the articles and screening was performed by two independent reviewers (MU and PD) following the inclusion criteria. Disagreements were resolved by consensus. The results of the search and the study inclusion process are reported in full and presented in a PRISMA-ScR flow diagram (Fig. 1) [29].

General information of the studies and the variables of interest were extracted using a data-charting form, including study design, sample size and composition, inclusion criteria, long COVID definition used, prevalence and symptoms, outcomes, and job performance (return to work, work ability, and measurement tools used). The form was developed by the research team based on JBI guidance and iteratively refined during the review process. Prior to full extraction, both reviewers pilot-tested the form on a sample of studies to ensure consistency in interpretation of variables and completeness of captured information. Adjustments were made through discussion until agreement was achieved. Data charting was then conducted independently by the two reviewers, with discrepancies resolved by consensus.

Quality assessment of the selected articles was performed using the Mixed Methods Appraisal Tool (MMAT) 2018, which allows for the appraisal of different categories of studies. As recommended by authors, detailed ratings are presented for each criterion to thoroughly inform study quality (Table 2). Two authors (PD and MU) independently assessed the quality of included reviews. Consistent with scoping review methodology, study quality was not used as a basis for exclusion. Instead, the appraisal informed the interpretation of findings, allowing the reviewers to consider the strength and potential limitations of the available evidence when identifying research gaps and implications for practice.

## Results

A total of 284 studies were identified. After removing duplicates and adding 8 articles identified through other sources, 217 articles were excluded based on title and abstract screening. After full-text assessment of 68 studies, 49 articles were excluded with reason (Fig. 1). This left 19 articles for inclusion: 15 from Europe, one from Latin America (covering 21 countries), one each from Egypt, Turkey and Thailand. Methodologically, one study used a qualitative approach, one employed a mixed method approach and the remaining 17 utilized quantitative methods, primarily cross-sectional designs (11 studies) as studies relied on surveys. A detailed summary for the findings can be found in Table 1. Quality appraisal can be found in Table 2, showing several issues related to the samples not being representative of the target population and the risk of response bias in cross-sectional studies.

**Table 1:** Long COVID characteristics, job performance assessment and outcomes in studies measuring job performance in healthcare workers with long COVID

Author & year (reference)	Study year & Country	Study design, Sample size and composition	Inclusion criteria	Long COVID definition	Long COVID prevalence & Symptoms	Job Performance Outcomes		
						Return to Work (RTW)	Work Ability (WA)	Tool
Bland et al. (2024) [35]	2022–2023 UK	Cross-sectional with *n* = 603 consultants 34% junior doctors 16% salaried GPs 12% GP partners 12%	UK-based medical doctor experiencing one or more post-acute COVID complications	NICE	Only partially or not recovered 62% One or two symptoms 25% Three or four symptoms 37% Five or six symptoms 20% Seven or more 18%	Respondents working full time when infected (*n* = 286, 57%) was greater than at survey completion (*n* = 156, 31%). Respondents who reported being unable to work due to long-term sickness *n* = 91, 18%.	Respondents well enough to return to work or train were most likely to work reduced hours or duties (*n* = 186; 40%), with females more likely than males to report an extended phased return (45%, *n* = 106 vs. 28%, *n* = 20) and occupational medical assessments (43%, *n* = 101 versus 33%, *n* = 24)	Survey
Dennis et al. (2023) [36]	2020– 2022 UK	Cohort with *n* = 536 HCWs = 32%	Individuals (patients and HCWs) recovered from acute COVID-19, but with ongoing symptoms.	Continuing symptoms for 12 weeks.	Systemic: 2% Cardiopulmonary: 1% Severe breathlessness: 30%Cognitive dysfunction: 38%Poor HRQoL: 45%	At follow-up, 95% of HCWs took time off work (median 180 days, range 41–308) and 63% had taken > 100 days off work. Those with ongoing symptoms took more time off work (median 172 days, range 42–300) compared to those whose symptoms had resolved (median 54 days, range 21–120)	NR	Health-related quality of life (HRQOL)
Fouad et al. (2022) [37]	2020 Egypt	Case control with *n* = 138 Nurses (39.1%) Workers (30.4%) Physicians (20.3%) Others (10.1%)	HCWs with positive (Cases) and negative (controls) PCR	-Post-acute COVID-19: symptoms extending beyond 3 weeks -Chronic COVID-19: Symptoms extending beyond 12 weeks	Prevalence 24.6% Dyspnoea 15.9%, Joint/Muscle Aches 14.5% Fatigue 14.5% Palpitation 14.5%	NR	HCWs with post-acute COVID-19 had significantly higher functional limitation. Despite having lower month/year work performance and relative presenteeism, the results didn’t reach statistical significance.	WHO and Work Performance Questionnaire; Post-COVID-19 functional status questionnaire
Foulkes et al. (2024) [38]	2022 UK	Cross-sectional *n* = 5053 Nursing 34% Administrative/Executive (office based) 16% Doctor 13% Healthcare Assistant 6% Physiotherapist/Occupational Therapist/SALT 5% Healthcare Scientist 4% Student 3% Midwife 2% Pharmacist 2% Estates/Porters/Security 2% Other 14%	SIREN participants were eligible if they had a recorded SARS-CoV-2 infection by 12 September 2022 and had not withdrawn from the study before the survey date. A recorded infection was defined as either a PCR positive sample, an antibody positive sample before vaccination, or an anti-N positive sample.	WHO and NICE 3-month criteria	1° infection 32.7% 2° infection 21.6% 3° infection 21.6% The three most frequently reported persistent symptoms across first, second and third infection episodes, respectively, were: general fatigue (17.3%, 12.8%, 10.8%), tiredness (16.9%, 10.7%, 8.2%) and shortness of breath (9.5%, 6.5%, 8.1%)	Reduced work hours 1° infection 8.9% 2° infection 15.8% 3° infection 27.3% Sick days Median [IQR] 1° 14 [7–50] 2° 7 [5–51] 3°10 [10] Mean (Range) 1° 56.8 (1−630) 2° 43.8 (3−196) 3° 10 (10−10)	Reduced ability to carry out work-related activities 1° Infection: • A lot (14.4%) • A little (42.1%) 2° infection: • A lot (17.3%) • A little (45.2%) 3° infection: • A lot (12.5%) • A little (68.8%) Changed work pattern 1° 13.9% 2° 20.1% 3° 38.5%	Online Survey
Grazzini et al. (2022) [39]	2020– 2021 Italy	Cross-sectional with *n* = 427 Nurses (43.6%) Physicians (11.9%) Healthcare Assistants (24.4%) Residents physicians (12.6%) Other (7.5%)	HCWs who tested positive to COVID-19 from March 2020 to April 2021	Symptoms persistent 3 months after recovering from the SARS-CoV-2 infection	Prevalence 34.4% Nurse 40.2% Healthcare assistants 46.6% Physicians 18.4% Other 28.1% widespread pain 21% asthenia 14% difficulty breathing 19%brain fog 8% Change in sleep pattern 37.2%Females (*p* = 0.001), older workers (*p* < 0.001), and healthcare assistants (*p* < 0.001) were more likely to report persistent symptoms.	NR	68.3% reported no changes in workload 15% reported an increase 7.7% reported a decrease Female workers perceived worse physical and mental health status than their male counterparts The nurses group reported increase in workload (*p* = 0.03)	Short Form health survey ISTAT (Italian National Statistical Institute)
Havervall et al. (2021) [40]	2020– 2021 Sweden	Cohort of *n* = 1395	HCWs who had mild COVID-19	At least 1 moderate to severe symptom lasting for at least 2 months At least 1 moderate to severe symptom lasting for at least 8 months	>4 months Any symptom 21.4% Fatigue 6.8% >8 months Any symptom 14.9% Fatigue 4.0%	NR	8% reported that their long-term symptoms moderately to markedly disrupted their work life 11% reported disruption in any Sheehan Disability Scale category as well as having at least 1 moderate to severe symptom lasting for at least 8 months.	Sheehan Disability Scale
Kaplan et al. (2022) [41]	2021 Turkey	Cross-sectional of *n* = 121 Physician (52.1%) Nurses (24.8%) Other (23.1%)	HCWs who had symptomatic diseases that are proven using RT-PCR and were diagnosed at least 12 weeks ago	Symptoms that last for >12 weeks	Prevalence 31.4% Fatigue 8.6% Attention deficit and concentration disorder 7.4% Dyspnoea 6.6% Sleep disturbance 5.7% 83% described dyspnoea that increased when climbing the stairs. It did not prevent them from doing their daily activities. No statistically significant correlation was found between age distribution, BMI, occupational group, and symptoms that lasted for >3 weeks	72.5% said they could carry out their daily work 12.5% spent < 50% of the day in bed 7.5% spent > 50% in bed	-EQ-5D-5 L 33.1% had worse health than before 59.1% health the same as before -Mobility No problems 76% Slight problems 20.7% Moderate problems 3.3% -Usual activities No problems 85.1% Slight problems 11.6% Moderate problems 3.3%	EuroQoL five-dimension (EQ-5D-5 L)
Ladds et al. (2021) [42]	2020 UK	Qualitative with *n* = 43 individual interviews=11 Focus groups=32	HCWs from online long COVID support groups and social media	Persistent symptoms lasting longer than 3 weeks	NR	individuals had good insight into their physical and mental limitations and expressed fear of coming back to work too soon, risking reputational damage or medico-legal ramifications.	Participants emphasised their strong work ethic and teamworking role and feared colleagues would perceive them as ‘shirkers’.	Groups and interviews
Lulli et al. (2023) [43]	2020– 2021 Italy	Cross-sectional with *n* = 318 Nurses (44.7%) Physicians (11.6%) Healthcare assistants (21.4%) Resident physicians (11.6%) Others (10.7%)	HCWs infected with COVID-19 and who tested positive for SARS-CoV-2 infection via a RT-PCR nasopharyngeal swab	Symptoms that persist after having tested negative via RT-PCR nasopharyngeal swab and persisting at the time of a medical examination, i.e., between 4 to 8 weeks after recovery.	Prevalence 56.3% Fatigue 32.1% Musculoskeletal pain 13.6% Dyspnoea 13.2% Older workers are more likely to experience post-COVID-19, especially musculoskeletal pain (*p* < 0.001), dyspnoea (*p* = 0.030), and fatigue (*p* = 0.011). No difference was found between the male and female workers.	NR	*Limitation in work activities* Any Symptom 18.1% Dyspnoea 9.5% Fatigue 29.3% Musculoskeletal pain 11.4% Limitations in work activities was independently linked to post-COVID-19 symptoms, particularly dyspnoea and fatigue.	Clinical examinations performed by Occupational Physicians in accordance with a specific protocol at the Occupational Medicine Unit
Martinez et al. (2021) [44]	2021 Switzerland	Retrospective cohort with *n* = 260 Nurses (47.3%) Medical staff (14.6%) Diagnostic staff (5.4%) Therapeutic staff (2.7%) House staff (5.8%) Administrative staff (14.2%) Other (10.0%)	HCWs who had a SARS-Cov-2 infection between 1 March 2020 and 15 April 2021 with a symptom persistence of >90 days.	Symptoms 90 days after COVID-19 diagnosis	Prevalence 26.5% Fatigue 68.9%* General weakness 46.7%* Concentration problems 44.4%* Breathing problems 42.2%**out of 47 who reported symptomsDepression or history of state of exhaustion (OR 4.36, 95% CI 1.64–10.56), older age and any lung disease (OR 3.80, 95% CI 1.70–8.49) were associated with LC.Gender, occupation and comorbidities did not show a significant association with LC.	1412 cumulative missed workdays (median 15, IQR 10–21, no missing data).	NR	online self-reported survey
Mendola et al. (2022) [45]	2020– 2022 Italy	cross-sectional with *n* = 56 Nurses (41.1%) Physicians (33.9%) Nursing assistant (17.9%) Other sanitary workers (3.6%) Non-sanitary workers (3.6%)	HCWs that required hospitalization for COVID-19	WHO: Persistence of signs and symptoms related to COVID-19 infection for more than twelve weeks and not explained by an alternative diagnosis.	Cough 77% Resting dyspnoea 84% Exertional dyspnoea 41% Arthromyalgia 42% Chest pain 82% Tachycardia 42% Asthenia 54% Cephalgia 56% Loss of memory 24% *Reported at 10 months	63% HCWs returned to work within six months since the infection Average time of 172.3 ± 149.3 SD days Full fit to work 39.1% Temporary not fit 2.2% Fit with restrictions 58.7% Restrictions related to physical exertion and night shift. No significant differences were found by age, gender, clinical history, and job category.	WA score improved from a median of 8 (5.25–10) to 9 (8–10), *p* < 0.05. All HCWs reach high scores except for nursing assistants resulting in a median of 8 (6.25–9.75). HCWs referred to a mean time to reach the current level of perceived WA of about 223 days.	WA from 0 to 10, considering before infection as 10
Müller et al. (2023) [46]	2022– 2023 Germany	Cohort with *n* = 127; HCWs 89 (70.0%) Nursing staff (55.9%) Medical staff (5.5%) Paramedical staff (4.7%) Therapeutic staff (3.9%) non-healthcare workers (30%)	HCWs and non-HCWs at post-COVID rehabilitation program	NICE	Exercise intolerance 100% Neurological ailments 98% Fatigue 91% Chest pain 91% There was a decrease in the prevalence of all symptoms at T2 except for fatigue.	mean 135.37 days (6–544) [min-max]	*Occupational inability* T1: HCWs (*n* = 89): 69.8% T2: HCWs (*n* = 87): 73.3% *Work Ability* T1: 74.0% poor; 26.0% moderate T2: 69.8% poor; 29.3% moderate; 0.9% good *beginning (T1) and end (T2) of inpatient rehabilitation No significant difference was observed between sex, professions, and severity of acute COVID-19 according to the WAI	work ability index (WAI)
Nehme et al. (2023) [47]	2021 Switzerland	cohort *n* = 3083 follow-up *n* = 900 Nursing staff 33.1% Physicians 14.8% Administrative staff 17.2% Other therapists and healthcare professionals 7.2%	All staff of the Geneva University Hospitals (HUG). The definition of healthcare workers included all hospital staff	NR	Symptom 68.4% at follow-up. The main symptoms were fatigue, headache, insomnia, cognitive impairment, stress/burnout, pain, digestive symptoms, dyspnea and cough.	More than 10 days of absenteeism 49.2% 19.7% of them attributed their absence from work to post-COVID symptoms	Functional impariment 23.9% Proffesional domain 35.1 (31.4–38.9)	Sheehan disability scale
Peters et al. (2022) [48]	2020 Germany	Cross-sectional with *n* = 2053 Nursing staff (60.4%) Medical staff (9.8%) Therapeutic staff (5.9%) Housekeeping (5.5%) Social service (4.3%) Administrative staff (4.2%) Other (9.9%)	HCWs who are insured with a suspected occupational COVID-19 infection before 31 December 2020	NICE: Persistent signs and symptoms after an acute SARS-CoV-2 infection that cannot be justified by an alternative diagnosis are classified into either long-COVID (four weeks or longer) or post-COVID (more than 12 weeks)	Prevalence: 72.8% Fatigue/exhaustion 82.9% Concentration/memory problems 70.7% Shortness of breath 56.5%Headache 41.3%	Until the survey, 5.2% of the participants (*n* = 107) had not returned to work	*Before COVID-19* No symptoms 9.3 ± 1.3 PCS 9.3 ± 1.2 *At the time of the survey* No symptoms 8.9 ± 1.7 PCS 6.8 ± 2.2 Risk factors: Age(OR 1.5; 95% CI 1.1–2.1), female sex (OR 1.6; 95% CI 1.2–2.2), pre-existing illness (OR 1.7; 95% CI 1.3–2.1), number and severity of symptoms during the acute infection phase (OR 1.2 and 1.6).	work ability index (WAI)
Saade et al. (2024) [49]	2022 France	Cross-sectional *n* = 1062 Administrative staff 12% Cleaners – Stretcher-bearers 6% Auxiliary Nurses 15% Nurses/Midwives/physiotherapists 32% Medical staff 8% HCWs with patient contact 17% HCWs without patient contact 3% Others 8%	All adult HCWs working at Rennes University Hospital	WHO	Prevalence 10% (95% CI, 9–12)	Initial sick leave after acute COVID, and sick-leave extension, were longer in HCWs who developed post-COVID, with medians of, respectively 7 [7–9] versus 7 [5–7] days, and (7 [5–12] vs. 6[0-7] days; *p* < 0.001 for both On return-to-work, 84% had residual symptoms, primarily asthenia/fatigue (72%) and cognitive impairment (25%).	NR	Survey
Tajer et al. (2023) [50]	2021 Latin America	Cross-sectional with *n* = 4673 Physicians (67,5%) Nurses (11,5%)	HCWs who have suffered from COVID-19 confirmed by PCR.	WHO: more than 3 months with persistence of at least two months	Fatigue 67% Insomnia 44.2% Anxiety 42.3% Myalgia 0.41.9%	16% reported requiring a modification of the work area: 12% asymptomatic 11,5% mild symptoms 18% moderate w/o hospitalization 25% hospitalized	Total recovery of activity: 76.6% almost total 15.6% only partial 5.7% not recovered 2.1% Age, number of symptoms and hospitalization associated with lack of recovery. Sex was not significantly associated	online survey
Tangsathajaroenporn et al. (2024) [51]	2022 Thailand	cross-sectional *n* = 609 Nurses	Nurses who had a history of COVID-19 infection 4 weeks before the survey, were 18 years or older, understood Thai, and willingly participated.	Individuals who experienced at least one abnormal symptom during the COVID-19 infection period, newly developed symptoms that persisted for at least 4 weeks, or symptoms that occurred more than 4 weeks after the infection.	Long COVID 26,3% Symptoms of fatigue (16.8%), cough (14.1%), and hair loss (12.2%	NR	Higher proportion of work limitations (37%) and a higher rate of job modifications (25,3%) *WAI* Poor 0,6% Moderate 18,2% Good 51,9%	WAI
Tempany et al. (2021) [52]	2020 Ireland	cross-sectional with *n* = 1176 known COVID-19 = 143	HCWs with COVID-19 at least 12 weeks prior to the study.	NICE: Signs and symptoms that (i) develop during or after an infection consistent with SARS-CoV-2 (ii) continue for >12 weeks (iii) are not explained by an alternative diagnosis	*PCR+* Prevalence 71% Fatigue 56% Sleep disturbance 40% Cognitive impairment 25% Psychological symptoms 22% Other physical symptoms 22% *Antibody+* Prevalence 23% Fatigue 14% Sleep disturbance 14% Cognitive impairment 6% Psychological symptoms 5% Other physical symptom 8%	NR	The subjective extent of recovery range 30–100%, median 88%, mean 82% (95% CI 79–85%). 19% reported feeling 100% recovered 22% reported feeling less than 70% recovered	survey
Torrance et al. (2024) [53]	2021–2022 Scotland	Mixed-methods study, online survey and qualitative interviews n = 471 Nurse and midwives 48% Doctor 8% Allied Health Professional 11% Ancillary 11% Administrative 17% Other 6%	Employed in a healthcare setting in NHS Scotland; aged ≥ 18 years; and self-reported LC signs and symptoms that continued or developed after acute COVID-19. This included both ongoing symptomatic COVID- 19 (from 4 to 12 weeks) and post-COVID-19 symptoms (12 weeks or more). A positive test was not a requirement for participation as these were not available to all HCWs early in the pandemic.	NICE & WHO	Duration of LC symptoms 1–3 months 9% 3–6 months 13% 6–12 months 30% More than 12 months 48% The mean number of symptoms reported was 9.5 (SD 5.2), including fatigue (88%), ‘brain fog’ (79%), breathlessness (69%), sleep disturbance (54%) and heart palpitations (48%)	18% (*n* = 79) of participants were unable to work at the time of the survey because of LC-related health problems.	A third (33%) reported changes to work duties. Most (80%) had not changed work hours, 17% had reduced their hours. Fatigue and cognitive issues (poor concentration, brain fog) most significantly inhibited return to work. Key to their ability to get back or remain at work were: nature and severity of symptoms; availability of workplace supports (especially flexibility); specific demands and expectations of roles; and own self-management/coping strategies	

NR: Not Reported; HCWs: Healthcare workers; LC: Long COVID; PCS: post-COVID; WHO: World Health Organization; NICE: National Institute for Health and Care Excellence; RTW: Return to work; WA: work ability; WAI: Work Ability Index; PCR: Polymerase chain reaction

**Table 2:**
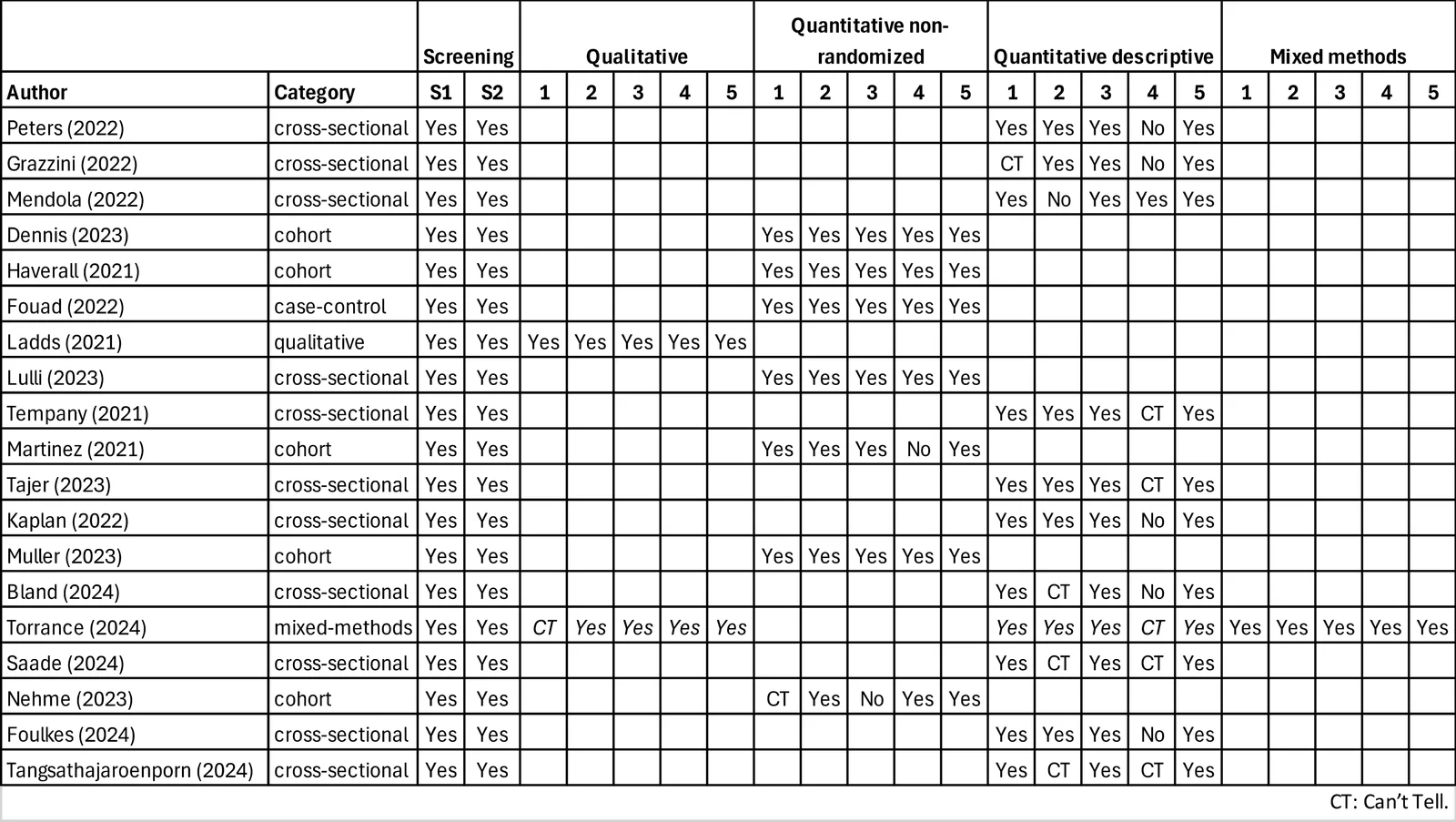
Quality appraisal of selected studies using the Mixed Methods Appraisal Tool (MMAT)

### Definition of long COVID

**Fig. 2:**
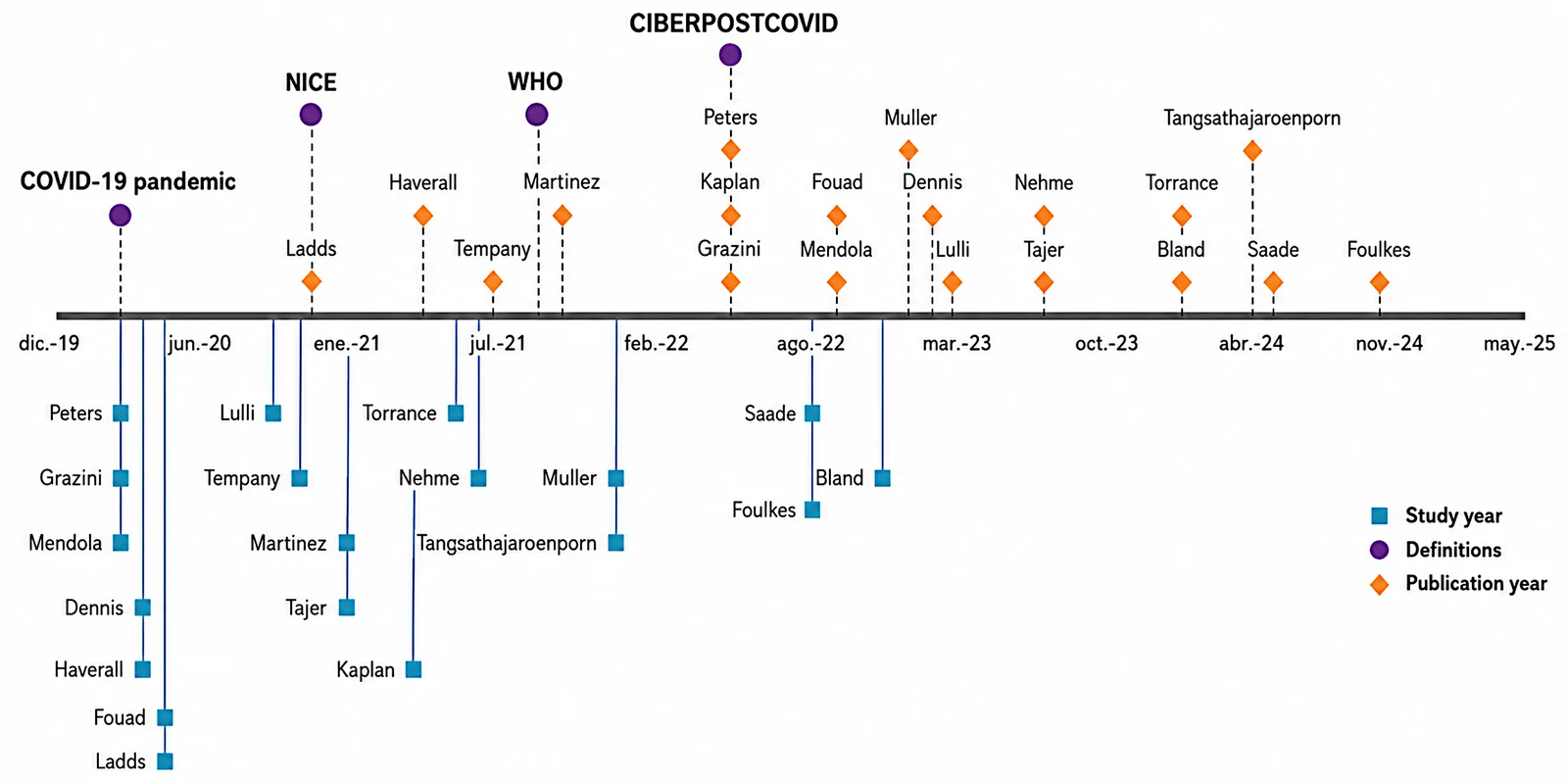
Timeline diagram of published studies assessing job performance in healthcare workers with long COVID, including their implementation years, the publication year and emerging definitions

Nearly all studies included a prior COVID-19 diagnosis as criterion. The most common definition of long COVID involves symptoms persisting for more than three months post-inflection. Notably, nine studies began before any official definition [36, 37, 39, 40, 42, 43, 45, 48, 52], while six adopted the NICE definition [38, 41, 44, 47, 50, 53] and five used the WHO definition [35, 38, 46, 49, 51] (some considered both). Only one study was published prior to these definitions (Fig. 2). The NICE definition was the first broadly adopted, appearing in six studies [35, 38, 46, 48, 52, 53], while the WHO definition was used in five [38, 45, 49, 50, 53], with other studies omitting or modifying key criteria such as excluding alternative diagnosis.

### Occupational groups represented

Most studies focused on physicians and nurses [35, 37, 39, 41, 43–51, 53], though seven studies also considered non-medical workers [38, 39, 44, 45, 47–49, 53]. Factors associated with higher impact in their job performance and prevalence included roles such as healthcare or nurse assistants (who experienced more work restrictions and lower work ability in two studies [39, 45]), older age, depression, pre-existing illnesses and greater severity of acute COVID-19 symptoms [38, 39, 44, 45, 48–50].

### Prevalence

Prevalence of long COVID among HCWs varied widely across studies, ranging from 10% to 98% with nine studies reporting rates above 50%. But, as shown in Table 3, the population used also varied which affects the prevalence described. Considering only studies with samples of HCWs who had COVID-19, cohort or all hospital staff (13 studies), the prevalence varied from 10% to 74%, with five studies reporting rates over 50%. Diagnostic methods influenced the prevalence in one study, with 71% of those diagnosed via polymerase chain reaction (PCR) tests and 23% via antigen rapid test (ATR) affected [52]. Gender was inconclusive due to four studies findings no significant association [41, 43, 44, 46] and five studies finding that female gender correlated with longer persisting symptoms and higher impact in their job performance [35, 38, 39, 48, 49].

**Table 3:** Long COVID prevalence (%) reported by author and the population used

Population	Author	Prevalence
All hospital staff	Nehme	68%
Cohort ongoing symptoms	Dennis	48%
Had COVID-19	Tempany PCR	71%
	Tempany ART	23%
	Martinez (Males)	22%
	Martinez (Females)	28%
	Lulli	56%
	Kaplan	31%
	Grazzini	34%
	Fouad	25%
	Haverall	15%
	Saade	10%
	Peters	74%
	Tajer*	67%
	Foulkes 1st	33%
	Foulkes 2nd	22%
	Foulkes 3rd	22%
Has acute Post-COVID symptoms	Bland	62%
	Tangsathajaroenporn	26%
Patients of a Post-acute COVID clinic	Muller	98%
HCWs who Self-reported Long COVID	Torrance	93%
HCWs who were Hospitalized with COVID-19	Mendola	84%

*Tajer reported fatigue prevalence and not Long COVID

### Return to work (RTW) outcomes

Thirteen studies assessed the RTW outcomes, revealing a substantial impact on RTW for HCWs, with many experiencing prolonged absences and with significant variation in range of missed workdays [36, 38, 44, 46], and several facing indefinite absenteeism or requiring a modified work area to return [35, 38, 45, 48, 50, 53]. RTW outcomes also show partial recovery and ongoing limitations, as one study found that 63% of HCWs returned within six months, but 58.7% with physical restrictions (e.g. limited physical exertion and night shifts) while four had not returned after a year [45].

A reduction in full-time work was observed among medical doctors, with a drop from 57% at the moment of infection to 31% at the moment of the survey, with 18% of participants attributing this to long-term sickness [35]. It was also observed related to number of infections, with 8.9% of the first infection and 27.3% of the third infection requiring reduced work hours [38]. In two studies, around 19% of participants were unable to work due to long COVID-related health problems [47, 53]. HCWs also expressed apprehensions of returning to work too soon, citing concerns about reputational damage or medico-legal ramifications [42].

### Work ability and functional limitations

Work ability was assessed in sixteen studies, all of which reported reduced scores for HCWs with long COVID compared to their pre-infection levels or asymptomatic colleagues, with many facing reduced hours [35, 38, 53], functional limitations [37, 38, 40, 43, 46–48, 50, 51], change of work patterns [38], and psychosocial challenges [42]. Studies using the WAI [46, 48, 51] and similar scales [45] highlighted that the number of symptoms significantly correlated with decreased work ability. On average, HCWs took 223 days to improve and reach their current level of work ability [45].

### Organisational responses and accommodations

Functional impairments and work limitations were frequently reported. Measures to mitigate the impact of long COVID on job performance included modifications to work duties and environments, reduced working hour and specific workplace accommodations [35, 38, 41, 45, 50, 51, 53]. Five studies reported various levels of required modifications from 16% to 40% [35, 38, 50, 51, 53]. Functional impairments and work limitations were noted in two studies by 23,9 and 37% of HCWs [47, 51]. Reduced ability to carry out work-related activities was reported as “A lot’” in 14.4%, 17.3% and 12.5% in the first, second and third infection, and as “A little” in 42.1%, 45.2% and 68.8% in the first, second and third infection [38]. Only two studies provided details on RTW evaluations by occupational health teams [35, 45]. Recognition of long COVID as an occupational disease was limited to one study [46] and notably cited as a source of frustration for HCWs due to the lack of recognition, even though their exposure was work related [53].

One interview conducted study identified four key aspects influencing their job performance: the nature and severity of symptoms, availability of workplace supports, specific demands and expectations of their role, and their self-management/coping strategies [53].

## Discussion

This scoping review provides an overview of the existing literature in the impact of long COVID on the job performance of HCWs, focusing on their RTW and WA. The 19 studies reviewed consistently demonstrate that long COVID affects the job performance of HCWs, with many unable to return to their pre-infection duties and/or requiring workplace accommodations. The significant variations in findings related to definitions, prevalence, job performance, and measures implemented to support HCWs with long COVID emphasize the ongoing challenges that this health condition presents in healthcare environments. Taken together, these findings indicate that long COVID is not only a clinical issue but also a workforce and service-delivery challenge for health systems.

The definition of long COVID has evolved during the pandemic, with most studies using criteria based on the NICE and WHO guidelines. However, inconsistences on how long COVID is reported across studies and settings remain a challenge. For example, early studies often lacked clarity on whether symptoms were attributable to long COVID or other pre-existing conditions. These discrepancies in definitions and reporting practices may contribute to difficulties in fully understanding the scope of the issue, as well as in comparing results across studies. Consistent use of standardized definitions in future research, such as the WHO, would facilitate more comparable results and improve our understanding of the condition’s impact on HCWs. Standardization would also support compensation systems, occupational surveillance, and the planning of rehabilitation services.

This review reflects the same variability found in the literature regarding the persistence of symptoms. Given that long COVID has not been officially recognized as an occupational disease worldwide, many studies relied on self-reporting methods to identify cases, with only one study utilizing an evaluation by the Occupational Health department [45]. Therefore, the underdiagnosis of long COVID as a new condition could have significant long-term impacts on healthcare settings, potentially affecting workforce availability and patient care [54]. Without formal recognition pathways, HCWs may encounter barriers in accessing sick leave, rehabilitation, or workplace adjustments, which in turn may delay recovery and safe return to practice.

Although the data points to a high prevalence of long COVID across the healthcare workforce, with almost half (9/19) of the included studies exposing a prevalence higher than 50%, there is still a significant variability in its reporting, the population used and gaps in the understanding of how sociodemographic factors influence job outcomes with long COVID. Only older age and the severity of the acute symptoms were associated with the development of long COVID and its impact on job performance, as they were more likely to report ongoing health issues that affected their work, corroborating findings from previous studies [12, 55]. Interestingly, while some studies have suggested that female gender might be linked to long COVID [11, 12, 55, 56], the overall evidence in this review relating it to job performance was inconclusive. This may arise from methodological differences and varying sample characteristics, highlighting the need for more rigorous research to better understand gender-related impacts.

Some of the studies in this review highlighted differences in how various groups were affected. HCWs in physically demanding roles, such as healthcare assistants, appeared to face greater work limitations and reduced work ability compared to their colleagues in less physical demanding positions [38, 39, 45]. However, the lack of consistent findings across occupational categories and gender groups underlines the complexity of these associations. Better occupational classification and reporting standards would allow services to anticipate which professional groups may require longer recovery periods or tailored accommodations.

The review highlights the significant impact of long COVID on job performance among HCWs, particularly concerning RTW and WA. Among the thirteen studies that examined RTW, extended periods of absence were a common finding. The wide variation in reported absence days highlights the diverse ways in which long COVID can affect individuals, and the need to work associated with being a HCW, which translates to high presenteeism specially during the pandemic [20, 21]. While a common factor analysed, the time it takes to return to work does not seem to reflect the impact on the job performance. From a service-planning perspective, partial productivity and modified duties must therefore be considered alongside headcount when estimating staffing capacity.

Work ability assessments consistently demonstrated reduced scores for HCWs affected by long COVID, both when compared to asymptomatic colleagues or their own pre-infection levels. Persistent symptoms such as fatigue, cognitive difficulties and shortness of breath were constantly reported as barrier to resuming full duties. Fatigue was frequently cited as a major contribution to decreased WA, reported in this review and previous studies as affecting both physical and cognitive health [8, 13, 56, 57]. The negative association between neurocognitive impairments, fatigue and work ability highlights the need for targeted interventions to support HCWs in managing these symptoms [8]. This is consistent with broader findings in the literature, were long COVID has been linked to reduced job performance across sectors, but particularly in high-demand environments like healthcare. The inability of HCWs to fully return to work puts and additional strain on healthcare systems, potentially leading to workforce shortages. Therefore, health systems may therefore need structured, multidisciplinary RTW programs, integration between occupational health and management, and flexible staffing models that accommodate fluctuating capacity.

Few studies provided detailed insights into the specific workplace accommodations that were offered to support HCWs dealing with long COVID, such as modifications to work duties, reduced working hours and access to mental health support. However, details on the implementation and effectiveness of these measures were sparse, with only two studies providing information on occupational evaluations for RTW. This gap suggests a need for more comprehensive approaches to assess fitness for work and implement effective accommodations. Recommendations for reducing the impact of long COVID on HCWs include temporary workload reductions and the elimination of night shifts [53, 58]. Different state agencies suggest evaluations by occupational safety and health services and adaptive measures such as remote work, flexible hour and reduced physical and cognitive workload, with permanent disability as a last resort, but do not refer to HCWs [59–61]. Implementation of supportive measures such as graded RTW might be beneficial for HCWs who are physically or cognitively impaired by their symptoms. Nonetheless, without formal evaluation, policy makers lack evidence on which measures provide the greatest benefit or are most cost-effective for the healthcare sector.

### Gaps of knowledge

This review identified several gaps. There is a need for longitudinal studies, consistent definitions, broader geographic representation, and improved reporting of gender and occupational variables. Importantly, intervention studies assessing the effectiveness of organizational responses are largely absent, limiting the ability to translate research into practice.

Based on the result of this scoping review, we suggest that future research should consider the unique demands of the healthcare sector, clarifying whether gender, occupational category, and working conditions such as night shift are associated with the development of long COVID in HCWs, and how it impacts their job performance, assessed with standardized tools like the WAI. Further investigation into the global impact of long COVID on healthcare systems will also provide valuable insights into the long-term consequences of the pandemic.

### Strengths and limitations

The main limitation of the available literature is the methodological variability, which affects both internal and external validity and could introduce biases, as shown by the MMAT appraisal. Furthermore, objective assessment of job performance varied. The heterogeneity in study design, sample size, definitions of long COVID and population used across studies poses a challenge for drawing firm conclusions. In addition, despite comprehensive searches, relevant studies may have been missed, particularly unpublished or non-English/Spanish research. The rapid evolution of the field means that new evidence may have emerged after the search period. Additionally, scoping review methodology aims to map evidence rather than provide pooled effect estimates, which limits the ability to quantify impact.

However, this review has strengths, including its focus on how long COVID affects HCWs’ job performance, adherence to JBI methodology and PRISMA-ScR checklist, and independent assessment of all studies by two reviewers. It also includes all types of HCWs from all healthcare settings and available geographical locations, providing insights into the effects that the worldwide pandemic has had in all contexts and countries.

## Conclusion

Long COVID significantly affects healthcare workers’ job performance, delaying return to work and reducing work ability beyond the acute phase of infection. Variation in definitions and prevalence continues to limit comparability across studies. Older age and severity of acute illness appear influential, while evidence regarding gender and occupational category remains inconclusive. Importantly, many workers resume duties with persistent symptoms or restrictions, creating ongoing pressure on both staff well-being and service delivery. Future research should harmonize measures and identify effective strategies and accommodations tailored to different occupational roles to mitigate the long-term impact on healthcare systems.

## Data Availability

All data analysed during this study are included in this published article and its supplementary information files.

## Annex 1 – Search criteria in pubmed

((“long covid”[Title/Abstract] OR “persistent covid”[Title/Abstract] OR “long term covid”[Title/Abstract] OR “postCOVID”[Title/Abstract] OR “postcovid syndrome”[Title/Abstract] OR “post acute covid 19 syndrome”[MeSH Terms]) AND (“healthcare worker*”[Title/Abstract] OR “healthcare professional*”[Title/Abstract] OR “healthcare employee*”[Title/Abstract] OR “healthcare personnel”[Title/Abstract] OR “health care worker*”[Title/Abstract] OR “health care professional*”[Title/Abstract] OR “health care employee*”[Title/Abstract] OR ((“health personnel”[MeSH Terms] OR (“health”[All Fields] AND “personnel”[All Fields]) OR “health personnel”[All Fields] OR (“health”[All Fields] AND “care”[All Fields] AND “personnel”[All Fields]) OR “health care personnel”[All Fields]) AND “health care worker*”[Title/Abstract]) OR “health care professional*”[Title/Abstract] OR “health care employee*”[Title/Abstract] OR “health care personnel”[Title/Abstract] OR “health worker*”[Title/Abstract] OR “health professional*”[Title/Abstract] OR “health employee*”[Title/Abstract] OR “health personnel”[Title/Abstract] OR “health personnel”[MeSH Terms])) AND (alladult[Filter])

## Abbreviations

WHO: World Health Organization
HCWs: Healthcare workers
RTW: Return to work
WA: Work ability
JBI: Joanna Briggs Institute
PRISMA-ScR: Preferred Reporting Items for Systematic Reviews and Meta-Analyses extension for Scoping Reviews
NICE: National Institute for Health and Care Excellence
WAI: Work Ability Index
MMAT: Mixed Methods Appraisal Tool
PCR: Polymerase chain reaction
ART: Antigen rapid test
